# Clinical Evaluation of Creatine Kinase and Aspartate Aminotransferase for Monitoring Muscle Effort in Working Dogs in Different Simulated Fieldworks

**DOI:** 10.3390/ani11071879

**Published:** 2021-06-24

**Authors:** Giuseppe Spinella, Simona Valentini, Vincenzo Musella, Enrico Bortolotti, Mirella Lopedote

**Affiliations:** 1 Department of Veterinary Medical Sciences, University of Bologna, Ozzano dell’Emilia, 40064 Bologna, Italy; giuseppe.spinella@unibo.it; 2 Department of Health Sciences, University of Catanzaro, 88100 Catanzaro, Italy; musella@unicz.it; 3 Clinica Veterinaria San Michele, 38010 Grumo di San Michele all’Adige, Italy; enricobor@me.com (E.B.); mirella.lopedote@yahoo.com (M.L.)

**Keywords:** search and rescue activity, creatine kinase, aspartate aminotransferase, pulse rate, respiratory rate, rectal temperature, dog

## Abstract

**Simple Summary:**

Search and rescue dogs are increasingly involved in finding survivors during catastrophic events. The need to investigate the possible physical conditions that can lead to disabling pathologies is urgent. In this clinical study, muscular effort was investigated through the evaluation of two muscle enzymes: creatine kinase and aspartate aminotransferase. The results show that effective conditioning supports submaximal activity (about 20 min), without any specific muscular enzymatic alteration.

**Abstract:**

The clinical profiles of muscle biomarkers (Creatine Kinase–CK-and Aspartate Aminotransferase–AST) performed during training may help in determining the fitness level of dogs and their potentiality to perform specific activities. This study investigated the potential variations of physiological parameters and muscular biomarkers in trained search and rescue dogs during search activity in two different areas. The aim was to verify the absence of any muscular enzymes after 20 min of search activity. The variations of physiological parameters (pulse rate; respiratory rate; rectal body temperature) and skeletal muscular biomarkers (CK and AST) were evaluated before and after search activity. Twenty-three trained dogs met the inclusion criteria and were divided into two groups. One group experienced search activity in a well-known area, while the second one in a similar, but unknown, area. The results for physiological parameters and skeletal muscular biomarkers values showed no significant differences between the two groups (*p* > 0.05), confirming that an effective conditioning protects against enzymatic alteration during a 20 min duration of submaximal activity.

## 1. Introduction

Search and rescue dogs (SRDs) have a great social impact due to their invaluable help in finding survivors during catastrophic events, such as earthquakes. Specific skills, such as obedience, handler bonding and human airscent detection are fundamental peculiarities of SRDs, but these athlete dogs also require adequate physical fitness to cover large areas of land when searching, often in adverse conditions [1,2]. In veterinary sports medicine, it is of great relevance to monitor canine training and conditioning to avoid anomalous skeletal muscular response, likely a prodromal state for the onset of muscle fatigue. Muscle fatigue is defined as a decrease in maximal force or power production in response to contractile activity [3]. Fatigue can originate at different levels of the motor pathway and is usually divided into central and peripheral components. Muscle fatigue is a commonly experienced phenomenon that limits athletic performance and other strenuous or prolonged activity [4]. However, the level of change in physiological, haematological and metabolic parameters occurring with exercise are dependent on several factors that include the typology of work and the grade of canine training as well as environmental factors [5]. The clinical profiles of muscle biomarkers performed during training may help in determining the fitness level of dogs and their potentiality to perform specific activities [5]. The most investigated biomarkers have been creatine kinase (CK), aspartate aminotransferase (AST), myoglobin and serum lactate concentrations. In veterinary sports dogs and SDRs, physiological parameters have been widely investigated, but scientific information on skeletal muscle biomarkers has been reported less frequently, generally in racing greyhounds or sled dogs, and less frequently, in SRDs [1,2,5,6,7]. Generally, a mild increase of specific muscle biomarkers was observed in dogs after prolonged endurance exercise and high-intensity racing. Longer and/or more intense exercise usually leads to an increase in significant changes, while less intense sessions may not have a significant impact. However, in endurance dogs, a targeted training programme may correctly prepare the dog for competition without any skeletal muscle injury [5]. To the authors’ knowledge, no publication has investigated the skeletal muscular response during submaximal exercise (20 min of duration) in SRDs, comparing the search activity in known or unknown working areas.

The aim of this study was to verify any potential variations of physiological parameters (pulse rate–PR; respiratory rate–RR; body temperature–BT) and skeletal muscular biomarkers (CK and AST) in trained SRDs during search activity in two different areas. One group experienced a known space of search (previously used for dog training), while the other group searched in an unknown area approached for the first time by the dogs (unknown field).

Our hypothesis was that well-trained dogs do not experience any permanent muscular damage during a submaximal search exercise (about 20 min), regardless of whether approaching a known or unknown search area.

## 2. Materials and Methods

Ethical approval for this study was obtained from the Animal Welfare Committee of the University of Bologna, in accordance with Italian DL 26/2014 (Project ID 914).

A team of 24 SRDs was involved in this observational study. All animals were skilled in search and rescue activity and fed with a commercial food for adult dogs. All dogs were conditioned under a similar training approach: conditioning included one day per week of obedience activity and two days per week of search activity in a training field. Moreover, each day, the dogs went freely with their owners along a 6 km route. The inclusion criteria were the completion of the search activity and absence of clinical and blood biochemical alterations before the search activity.

### 2.1. Clinical and Laboratory Monitoring

All dogs were clinically monitored by two licensed doctors in veterinary medicine before the trial to ensure a good state of health and suitability to perform the scheduled search activity. No dogs received any medication or drugs within 15 days before the search activity. Dog signalments and physiological parameters (pulse rate, respiratory rate and rectal body temperature) were recorded for all dogs at rest, in order to exclude any physiological variation related to environmental factors and the potential excitement state. The pulse rate was detected with palpation of femoral artery and respiratory rate was measured by thoracic visual observation. A first blood sample for CK and AST evaluation was collected 15 min before search activity by saphenous vein. Immediately after activity, PR and RR were measured; a second blood sample and BT measurement were collected and recorded two hours after the activity. The timetable for the evaluation of muscular enzymes was scheduled two hours after activity in order to ensure that an increase of enzyme concentration could be reasonably attributed to prolonged muscle damage secondary to an excessive muscular effort [8].

### 2.2. Search Activity and Features of the Search Areas

The study was created following the regulations of the Italian National Canine Association (Ente Nazionale Cinofilia Italiano–ENCI). The test involved a search by the dog for two missing persons (‘victims’) in an area from 30,000 to 50,000 square m, depending on the morphology of the land and vegetation. The evaluation parameters for this test were: (a) docility, cooperation of the dog with the handler, prompt and targeted execution of orders, maintaining motivation in the search; (b) intensity in searching, behaviour during the search, temperament, motivation, enthusiasm, physical condition; (c) agility, movement in the search area, facing difficulties; (d) independence in searching; (e) the quality of the tactical choice and its implementation, management of the entire search operation; (f) behaviour of the dog with disruptive walker-ons; (g) finding the ‘victim’.

On the day of search activity, the team was randomly divided into two groups. Group 1 searched the well-known area, while Group 2 searched an unknown area. The start of the activity occurred approximately every 30 min for each dog. A maximum of 20 min of search time was allowed for each dog to locate the two ‘victims’ (as required by time imposed by ENCI regulation).

Area n.1 (area already known by dogs of Group 1)–wooded area in Clusone (North of Italy), east side, rich in larch and fir trees with a dense undergrowth with many brambles. Within the search area, there were some clearly visible paths, with the main part of the area located on a natural terrace at an altitude of 549 m above sea level, which then sloped down on the north side and on the west side to a flat area below. The total height difference of the area was about 50 m.

Area n.2 (area unknown by dogs of Group 2)–located within the pine forest of Clusone, but placed towards Mount Senda, on the west side. A steep, but not impervious, slope rose from 530 to 650 m altitude and the vegetation was composed of larches and firs with dense undergrowth, also characterised by some patches of brambles. The total difference in height of the area was about 120 m.

During the clinical study, the environmental air temperatures ranged from 18 to 26 °C with 70% humidity and a relative wind speed of 7–8 m/h.

### 2.3. Statistical Analysis

All data were submitted to descriptive (mean ± standard deviation and median) and analytic statistical analysis for the entire team and specifically, within Groups 1 and 2. All data were previously submitted to the Shapiro–Wilk test for an evaluation of normality distribution. If normal distribution was not reported, physiological and serum parameters were evaluated with the Wilcoxon–Mann–Whitney *t* test, in order to investigate any significant variation between rest and after the search activity. Significance for all tests was set at *p* < 0.05. Statistical analyses were performed using Stata software, version 15 (StataCorp, College Station, TX, USA, 2017).

## 3. Results

Twenty-four dogs were enrolled in this study and all dogs completed the search activity. However, one dog was excluded because of high pre-competition values of AST and CK. The 23 dogs were: five German Shepherds, three Malinois, three Golden Retrievers, three Labradors, three mixed breed dogs, two Border Collies, two Australian Shepherds, one Jack Russel terrier and one Weimaraner. Ten females and two males were included in Group 1, and nine females and two males in Group 2. The mean age of the entire team was 4.6 ± 2.5 years (median 5): 5.3 ± 2.4 and 3.9 ± 2.4 years, respectively in Group 1 and Group 2.

**Figure 1:**
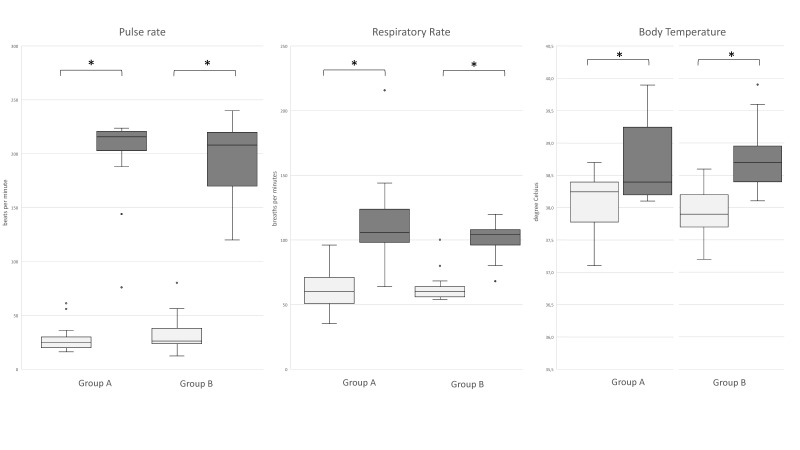
Boxplots of pulse rate, respiratory rate and body temperature (°C) monitored before and after activity, in Group 1 (**A**) and Group 2 (**B**). Statistical differences were observed in all three physiological parameters (* *p* < 0.05).

No significant difference was observed between the two groups for age distribution (*p* = 0.191). Mean PRs (beats per minute), respectively for Groups 1 and 2, were 61.6 ± 17.5 and 64.5 ± 13.8 at rest, and 114.6 ± 38.7 and 99.82 ±14.8 after activity. Mean RRs (breaths per minutes) were 29.7 ± 14.3 and 33.6 ± 19.8 at rest, and 197.3 ± 44.3 and 194.1 ±37.1 after activity, respectively for Groups 1 and 2. Mean BTs were 38 ± 0.4 °C and 37.9 ± 0.4 °C at rest, and 38.6 ± 0.6 °C and 38.8 ± 0.5 °C two hours after activity, respectively for Groups 1 and 2. For physiological parameters (PR, RR, BT), no significant differences were observed for variations between the two groups in relation to physical activity; while significant differences were shown for PR, RR and BT before and after activity (*p* < 0.01) for both groups (Figure 1).

**Figure 2:**
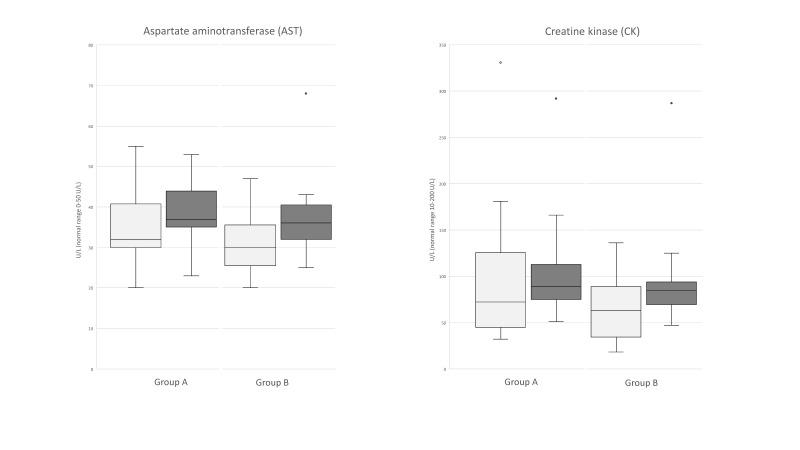
Boxplots of aspartate aminotransferase (AST) and creatine kinase (CK) monitored before and 2 h after activity in Group 1 (**A**) and Group 2 (**B**). No statistical differences were observed.

The mean AST values of 34.75 ± 9.2 U/L and 31.18 ± 8.5 U/L were reported, respectively for Groups 1 and 2 before activity, and 39.42 ± 8.5 U/L and 37.9 ± 11.5 U/L two hours after activity. Mean CK values of 104.33 ± 86.1 U/L and 66 ± 38.8 U/L were reported, respectively for Groups 1 and 2 before activity, and 107.42 ± 65.6 and 99.82 ± 65.3 two hours after activity. For AST and CK values, no significant differences were reported between the groups (*p* > 0.05) (Figure 2).

## 4. Discussion

This study aimed to investigate the absence of muscle damage in well-trained dogs during a submaximal search exercise (about 20 min), in two different working areas (known and unknown). The results show that no statistical differences were observed between the two groups and the hypothesis was therefore confirmed.

Both groups reported an increase of PR and RR immediately after activity as well as an increase in BT.

Variations in physiological parameters for working and sports dogs have been investigated during competitive and conditioning activity [1,2,5,6,7,8]. Pulse and respiratory rates generally increased after activity in response to increased energy production during exercise and to eliminate the produced heat as a by-product. However, if heart and pulse rates are measured immediately before a competition, a significant increase could be observed compared to rest, as a response to exercise anticipation, also reported as the ‘eureka effect’ [2,6,8,9]. Systematic training is a continuous process that aims to maximise aerobic capacity through weekly exercise sessions, individually prescribed to decrease the risk of musculoskeletal injury [10]. Our investigation showed a BT increase after activity, but this variation was within the physiological range two hours after the activity. Indeed, as previously reported by Matwichuk et al. (1999) in healthy Labrador retrievers, no significant rectal temperature variations were observed in exercising dogs when the environmental temperature ranged between 11–28 °C [11].

In our study, muscular enzymes results demonstrated a mild increase, but this variation was not statistically different and within the normal range.

Creatine kinase (CK) is the best indicator of striated muscle damage as most of its activity is in skeletal muscle. The increase in plasma CK activity in dogs is due to its leakage through the cell membrane and therefore, is evident in all conditions associated with muscle inflammation, necrosis or degeneration [12]. CK has a half-life of 2–3 h, so high serum activity after two hours could be indicative of recent or active muscle damage.

Aspartate aminotransferase (AST) is also indicative of muscle damage, although it is less organ-specific than CK. AST is also found in the liver. The AST assessment is useful, together with the CK assessment, to understand if muscle damage is evolving or resolving [12].

Previous studies on sports dogs revealed that prolonged endurance exercise and high-intensity racing induced a mild, but significant, elevation in the skeletal muscle biomarkers, CK and AST [13,14]. However, Cerqueira et al. observed that an endurance-training programme (approximately 42–56 min) improved the aerobic capacity of dogs without any skeletal or cardiac muscle injury [5]. Working dogs that experienced a maximum of 10 min of search activity for victims, after helicopter transport, reported an increase of CK and AST blood concentrations after search activity, but normal concentrations were detected after two hours from the end of the activity [8]. For this specific reason, our second blood collection was scheduled two hours after activity, to minimise the exercise effect on muscular metabolism and to detect potential muscular damage.

A study conducted by Hinchcliff reported that differences in serum biochemical variables between dogs that completed a race and those that were retired, were not clinically significant. It was therefore not possible to identify any consistent factors that were associated with a failure to complete a race (retired dogs) [15].

## 5. Conclusions

In conclusion, our study aimed to investigate whether prolonged muscle injury occurred following a submaximal search exercise (about 20 min) in well-trained SDRs that experienced a search activity in an unknown area, as normally happens in real conditions. All dogs correctly concluded their scheduled activity: potential variations of skeletal muscular biomarkers (CK and AST) were not observed two hours after the activity. Moreover, it could be assumed that the pause of two hours, after the end of the research activity, may be enough for observing the values of CK and AST in physiological ranges and consequently, the dog could be ready for a new work session. However, this latter hypothesis should be investigated with further prospective studies.

## Data Availability

All data is contained within this article. Interested qualified researchers may request further information by contacting the corresponding author.
